# Use of sodium nitroprusside in retrograde percutaneous coronary intervention for chronic total occlusion: A case report

**DOI:** 10.1097/MD.0000000000011498

**Published:** 2018-07-20

**Authors:** He Huang, Yao-Jun Zhang, Yong-Zhen Fan, Xi Wu, Christos V. Bourantas

**Affiliations:** a Department of Cardiology, Xiangtan Central Hospital, Xiangtan; b Department of Cardiology, Xuzhou Third People's Hospital; c Barts Health NHS Trust; d Department of Cardiovascular Sciences, University College London, London, UK.

**Keywords:** chronic total occlusion, coronary collaterals, retrograde recanalization, sodium nitroprusside

## Abstract

**Rationale:**

Chronic total occlusion continues to be a challenging lesion subset for percutaneous coronary intervention.

**Patient concerns:**

A 65-year-old male patient was admitted with symptoms of angina pectoris for 9 months.

**Diagnoses:**

Coronary angiography showed a severe stenosis in the proximal left anterior descending artery and a chronic total occlusion (CTO) in the proximal right coronary artery. The complexity of the CTO was stratified using the J-CTO score and the PROGRESS CTO score.

**Interventions:**

Antegrade wire escalation for CTO of RCA failed. The septal collaterals to RCA were initially judged to be poor and not suitable for intervention.

**Outcomes:**

However, administration of sodium nitroprusside improved collateral flow and enabled the identification of retrograde channels suitable for wire crossing and successful retrograde PCI.

**Lessions:**

The study showed that faintly visible to even invisible septal collateral connections can be crossed with the septal “trial and error” surfing technique after the administration of sodium nitroprusside.

## 1 Introduction

Percutaneous revascularization of chronic total occlusion (CTO) continues to be challenging. With the utilization of the retrograde recanalization approach through collaterals, the success rate has significantly improved.^[1]^ Over the past 10 years, experienced operators and technicians developed different retrograde techniques as well as new guidewires, microcatheters, and balloons dedicated to this approach. However, the success of this approach is highly dependent on coronary collaterals.^[1]^ To our knowledge, this is the first report of the use of sodium nitroprusside to improve collateral circulation leading to a successful retrograde percutaneous coronary intervention for CTO.

## 2 Case report

**Figure 1:**
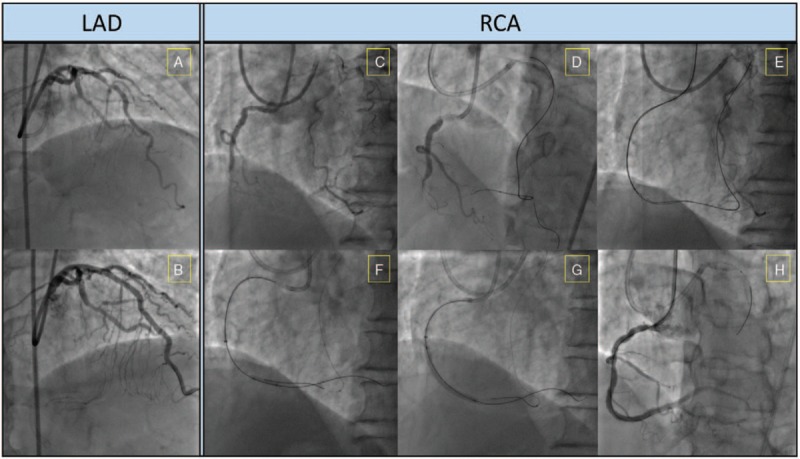
Interventional procedure of the reported case. (A) Severe stenosis presented in the middle LAD; (B) a good angiographic results was obtained following implantation of a DES; (C) CTO of the middle tract of right coronary artery (RCA); (D) a Sion guidewire was advanced in the distal RCA through the first septal collaterals; (E) kissing wire technique was used for recanalization of RCA-CTO; (F) Predilation was performed after antegrade crossing with a Conquest Pro 8-20 guidewire; (G) DESs were implanted successfully from the distal to the middle RCA; (H) Final angiography of RCA after revascularization with DES implantation. CTO = chronic total occlusion, DES = drug-eluting stent, LAD = left anterior descending artery, RCA = right coronary artery.

A 65-year-old male patient with a history of hypertension and hyperlipidemia was admitted with symptoms of angina pectoris in the preceding 9 months. An electrocardiogram revealed ST-segment depression in anteroseptal leads and pathological Q waves in inferior wall leads. An echocardiography determined a normal left ventricle cavity size with good systolic function and no any regional wall motion abnormalities (left ventricular ejection fraction 57%). Coronary angiography showed a severe stenosis in the proximal left anterior descending artery (LAD) (Fig. 1A) and a CTO in the proximal right coronary artery (RCA) (Fig. 1C). The complexity of the CTO was stratified using the J-CTO score (3; re-try lesion, blunt stump type, presence of calcification, and occlusion length ≥20 mm) and the PROGRESS CTO score (2; proximal cap ambiguity and absence of “interventional” collaterals).^[2]^

Firstly, the LAD artery was treated with a 3.5 mm × 18 mm biodegradable polymer drug-eluting stent (Fig. 1B). The RCA revascularization was then attempted employing a wire escalation strategy with the Fielder XT, Miracle 3 and 6 guidewires (Asahi Intecc., Nagoya, Japan), however without success. Hence a retrograde approach was initiated. The collateral connection from the left circumflex artery to RCA was avoided due to its severely corkscrew type tortuosity and the small collateral size (collateral connections 1, CC1). Consequently the septal 1 (S1) collateral channel was considered a viable option despite its diminutive size (CC class 0).

**Figure 2:**
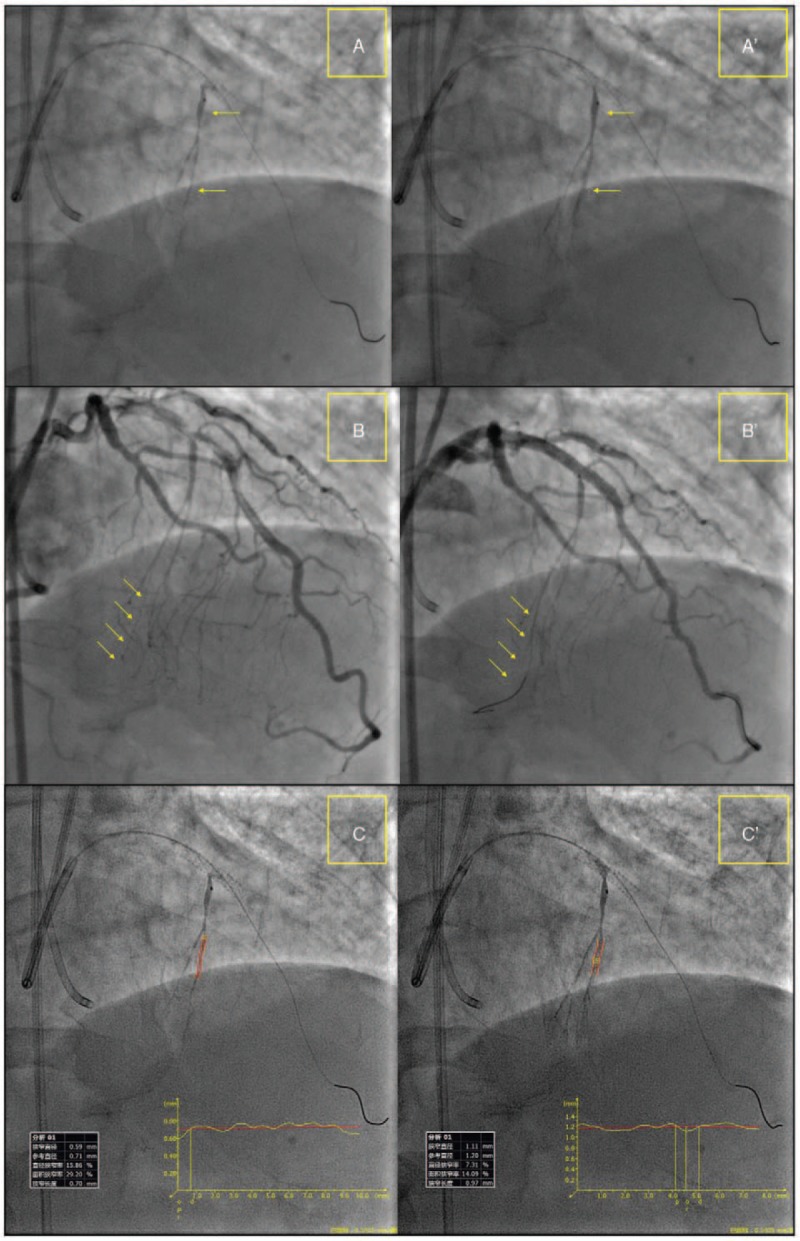
Coronary collateral channel pre- and post-selective injections of sodium nitroprusside. Septal collateral channel improved after selective injections of sodium nitroprusside by microcatheter (A, B, A′, and B′). Quantitative angiography analysis showed that mean reference vessel diameter of septal branch increased from 0.7 to 1.2 mm (C and C′).

A Sion guidewire (Asahi Intecc., Nagoya, Japan) was advanced into the S1 branch supported by a Finecross MG coronary microguide catheter (Terumo Medical Corporation, NJ). A tip injection failed to identify collateral flow from the S1 branch to the distal RCA (Fig. 2A). Hereafter sodium nitroprusside (100 μg) was selectively injected twice through the Finecross microcatheter. Repeated tip injections ensued that the collateral size significantly improved from CC 0 to CC 1-2, and enabled adequate visualization of the distal RCA (Fig. 2A′, B, and B′). Quantitative angiography analysis was performed using the QAngio XA analysis software (Medis Medical Imaging Systems Inc., Leiden, The Netherlands). The mean reference vessel diameter of the first S1 branch increased from 0.71 to 1.20 mm (Fig. 2C and C′). Accordingly, the Sion guidewire was advanced with no difficulty to the distal segment of the occluded RCA with the assistance of the Finecross microcatheter (Fig. 1D). Hereafter, a retrograde wire crossing technique with the Sion, Gaia First, Gaia Second, and Conquest Pro guidewires (Asahi Intecc., Nagoya, Japan) was attempted but failed. Thus, a kissing wire technique was used for the recanalization of the occluded RCA with a Conquest Pro 8-20 guidewire supported by a Finecross 130 mm microcatheter (Fig. 1E). Retrograde angiography confirmed that the antegrade wire was in the distal true lumen of the RCA. Finally, 2 biodegradable polymer sirolimus-eluting stents (2.5 × 33 mm and 3.0×36 mm) were implanted from the distal to the proximal RCA after predilation (Fig. 1F and G). After post-dilation, final angiography and intravascular ultrasound demonstrated that stents were well expanded with excellent stent strut apposition (Fig. 1H).

## 3 Discussion

CTO continues to be a challenging lesion subset. With advances in stent design and ancillary devices, and also the introduction of novel CTO recanalization techniques, through retrograde collaterals, procedural success has recently improved. Retrograde CTO PCI has been introduced and constitutes an effective alternative option for the treatment of complex CTO. However procedural success depends on the quality of coronary collaterals.^[3]^ However, a diminutive collateral channel size coupled with tortuosity of the collateral vessels are critical risk factors which may result in an increased rate of complications as well as failure of the retrograde PCI.^[4,5]^

Septal and epicardial CCs are 2 types of potential collateral channels for retrograde PCI. Septal CC is relatively safe and considered as default choice by the majority of the CTO operators. Size and tortuosity from its origin to the distal connection are the 2 most important characteristics to determine usability of CCs. CC0 and tortuous CC are more likely to be associated with failed retrograde recanalization. Recently, a few highly experienced operators have attempted to cross small CCs (specifically CC0 septal CCs) using a gentle “surfing” technique if there is distal filling of the collateral bed. Thus, size may be less of a determinant for successful septal CC wiring. On the other hand, a greater relative magnitude of branching could be a severe limitation to wire advancement. One may tackle this problem by selective injections from the microcatheter, albeit with an increased risk of septal rupture and septal hematoma, which make the septal CC unusable.

In this case report, there was no suitable retrograde channel. However, administration of sodium nitroprusside improved collateral flow and enabled the identification of retrograde channels allowing successful wire crossing and retrograde PCI in a patient with an unfavorable collateral channel. It has been learned that faintly visible to even invisible septal CCs can be crossed with the septal ‘trial and error’ surfing technique after the administration of sodium nitroprusside.

## Author contributions

**Conceptualization:** He Huang, Yong-zhen Fan.

**Data curation:** He Huang, Yong-zhen Fan.

**Formal analysis:** He Huang, Yong-zhen Fan, Xi Wu.

**Investigation:** He Huang.

**Methodology:** He Huang, Yao-Jun Zhang, Xi Wu, Christos V. Bourantas.

**Supervision:** He Huang, Yao-Jun Zhang, Christos V. Bourantas.

**Writing – original draft:** He Huang, Yao-Jun Zhang, Yong-zhen Fan, Christos V. Bourantas.

**Writing – review & editing:** He Huang, Yao-Jun Zhang, Yong-zhen Fan, Christos V. Bourantas.
